# Cancer-derived exosomes as novel biomarkers in metastatic gastrointestinal cancer

**DOI:** 10.1186/s12943-024-01948-6

**Published:** 2024-04-01

**Authors:** Danyang Zhong, Ziyuan Wang, Zhichao Ye, Yifan Wang, Xiujun Cai

**Affiliations:** 1 Department of General Surgery, Sir Run Run Shaw Hospital, Zhejiang University School of Medicine, Hangzhou, 310016 China; 2 National Engineering Research Center of Innovation and Application of Minimally Invasive Instruments, Hangzhou, 310016 China; 3 Zhejiang Minimal Invasive Diagnosis and Treatment Technology Research Center of Severe Hepatobiliary Disease, Hangzhou, 310016 China; 4 Key Laboratory of Laparoscopic Technology of Zhejiang Province, Hangzhou, 310016 China

**Keywords:** Exosomes, Metastasis, Biomarker, Gastrointestinal cancer

## Abstract

Gastrointestinal cancer (GIC) is the most prevalent and highly metastatic malignant tumor and has a significant impact on mortality rates. Nevertheless, the swift advancement of contemporary technology has not seamlessly aligned with the evolution of detection methodologies, resulting in a deficit of innovative and efficient clinical assays for GIC. Given that exosomes are preferentially released by a myriad of cellular entities, predominantly originating from neoplastic cells, this confers exosomes with a composition enriched in cancer-specific constituents. Furthermore, exosomes exhibit ubiquitous presence across diverse biological fluids, endowing them with the inherent advantages of non-invasiveness, real-time monitoring, and tumor specificity. The unparalleled advantages inherent in exosomes render them as an ideal liquid biopsy biomarker for early diagnosis, prognosticating the potential development of GIC metastasis.

In this review, we summarized the latest research progress and possible potential targets on cancer-derived exosomes (CDEs) in GIC with an emphasis on the mechanisms of exosome promoting cancer metastasis, highlighting the potential roles of CDEs as the biomarker and treatment in metastatic GIC.

## Introduction

According to the latest statistical data on cancer, 1,918,030 new cases and 609,360 cancer-related deaths (CRDs) had been documented. Among them, the GIC ranks second but is responsible for about 28% of cancer-related deaths. Colorectal cancer (CRC) is the most prevalent GIC which accounts for 44% of all cancer cases, followed by pancreatic cancer (PC) which has a 5-year survival rate of less than 11% and is the most lethal cancer [1]. Furthermore, global GIC morbidity and mortality continue to rise [1]. Metastatic cancers are in charge of 90% of CRDs [2, 3], and metastatic GIC is characterized by high aggressiveness and heterogeneity which take primary responsibility for death in patients with malignant GIC [4]. While diverse diagnostic methods, including gastroscope, computed tomography (CT) scan, and pathological examinations [4, 5], are vital for terminal-stage cancer with conspicuous symptoms, the main challenge in GIC diagnosis stems from the restricted sensitivity of these technical approaches to small lesions in pre-metastatic GIC or residual masses post radiotherapy and chemotherapy. In addition, some methods own limitations on incursion and potential of transmission. Current therapeutic targets and diagnostic biomarkers still do not meet the clinical needs of the goal of treatment for GIC. Presently, there is an urgent need for novel diagnostic methods with accurate detection rates and high-quality specificity, especially in cases of pre-metastatic PC.

As the liquid biopsy undergoes continual expansion and refinement, it was not until very recently that exosomes as the biomarkers for cancer metastasis detection have been appreciated. Exosome, initially elucidated in 1983 by Johnstone et al. [6, 7], is a subset of extracellular vesicles (EVs) with sizes ranging from 40 to 160 nm. They are ubiquitously secreted by nearly all cell types, with a particular emphasis on cancer cells [8]. Initially, researchers considered that exosomes are just the lipid bilayer to transport cellular metabolic waste [6]. However, our comprehension of exosomal function has been substantially deepened and broadened over the past decades. As research dug deeper, massive studies revealed that exosomes are essential for the intercellular communication from cancer cells to neighboring stromal and immune cells to promote tumor metastasis via mediating immune evasion, facilitating pre-metastatic niche (PMN) formation, angiogenesis, and epithelial-mesenchymal transition (EMT) [9–12].

In this review, we intend to present a comprehensive overview on how GIC-derived exosomes promote cancer metastasis through participating in PMN formation, immune evasion, angiogenesis, and EMT. Moreover, founded on the culmination of exosomes as discerning indicators within the context of liquid biopsy, we predominantly summarize the exosomes derived from CRC, hepatocellular carcinoma (HCC), gastric cancer (GC), and PC as pivotal prognostic, diagnostic, and predictive biomarkers for metastatic GIC.

## The mechanisms of CDEs-related cancer metastasis

In 1889, Stephen Paget proposed a promising and unprecedented hypothesis of metastasis which is “seed and soil” and revealed that the metastasis of cancer is not random but the interactions between 'seeds' (the cancer cells) and the 'soil' (the host microenvironment) [13, 14]. Several studies have intensively illustrated that the pending metastasis site could induce the formation of tumor-friendly microenvironment which are termed PMNs, to suit cancer cell growth [15]. In this process, the medium is exosomes, which means that exosomes carry different cargos to mediate cell-to-cell communication which can assist primary cancer in selectively modifying the target organs of future metastases. Meanwhile, exosomes can reach target organs to increase vascular permeability, remodel stromal cells and extracellular matrix (ECM), and change the immune system before metastasis occurs [14, 16–18].

**Fig. 1:**
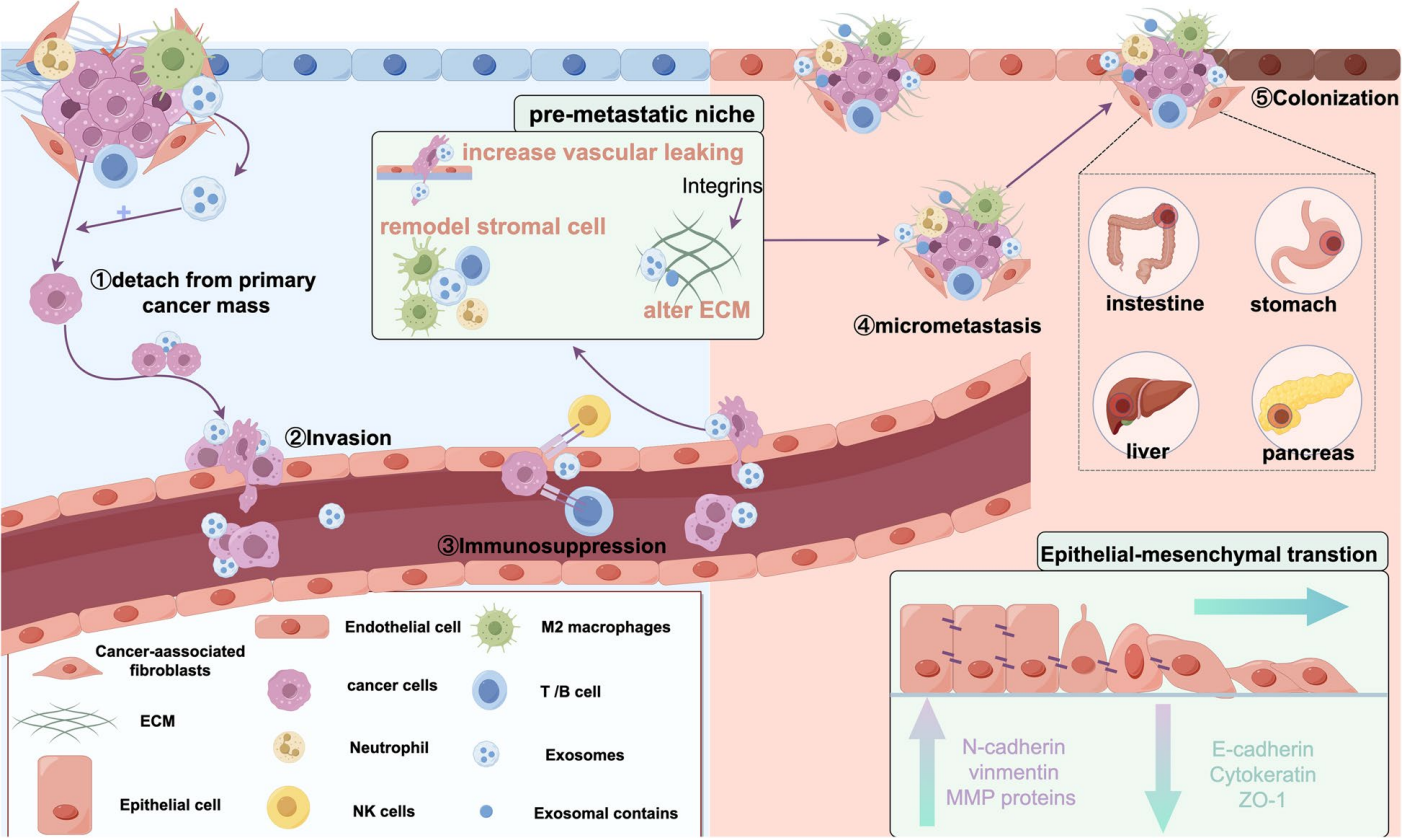
The mechanisms of CDEs in cancer metastasis. CDEs are involved in the processes of assistant tumors to depart from primary site, immune evasion, angiogenesis, increasing vascular permeability, the formation of pre-metastatic niche (PMN), epithelial-mesenchymal transition (EMT), planting on distant organs, and promoting cancer metastasis

Metastasis, also termed the metastatic cascade, is categorizable into three distinct and overlapping phases—dissemination, dormancy, and colonization [19]. And exosomes assume a pivotal role in the intricate process of tumor metastasis: ①Aided by CDEs, cancer cells detach from the primary cancer mass. ② CDEs can assist cancer cells in passing the bloodstream or lymphatic vessels to infiltrate surrounding or target tissues. ③CDEs induce immunosuppression and escape from immune surveillance by interaction with immune cells [20]. ④Exosomes carry a bunch of proteins, integrins (ITGs), RNA, and DNA to alter the ECM, re-organize the target organ structure and vasculature, recruit stromal cells to facilitate the target organ, and form the tumor-friendly microenvironment or PMNs. ⑤CDEs activate EMT to increase invasion ability and promote migration (Fig. 1).

These detailed and in-depth studies provide us with a comprehensive reference for understanding the role of exosomes in the metastasis, deterioration, and treatment resistance of tumors. However, the most pivotal questions that remain unresolved are how and to what extent the CDEs work in the process of cancer metastasis between the receptors and distinct sites.

### Exosomal biogenesis

**Fig. 2:**
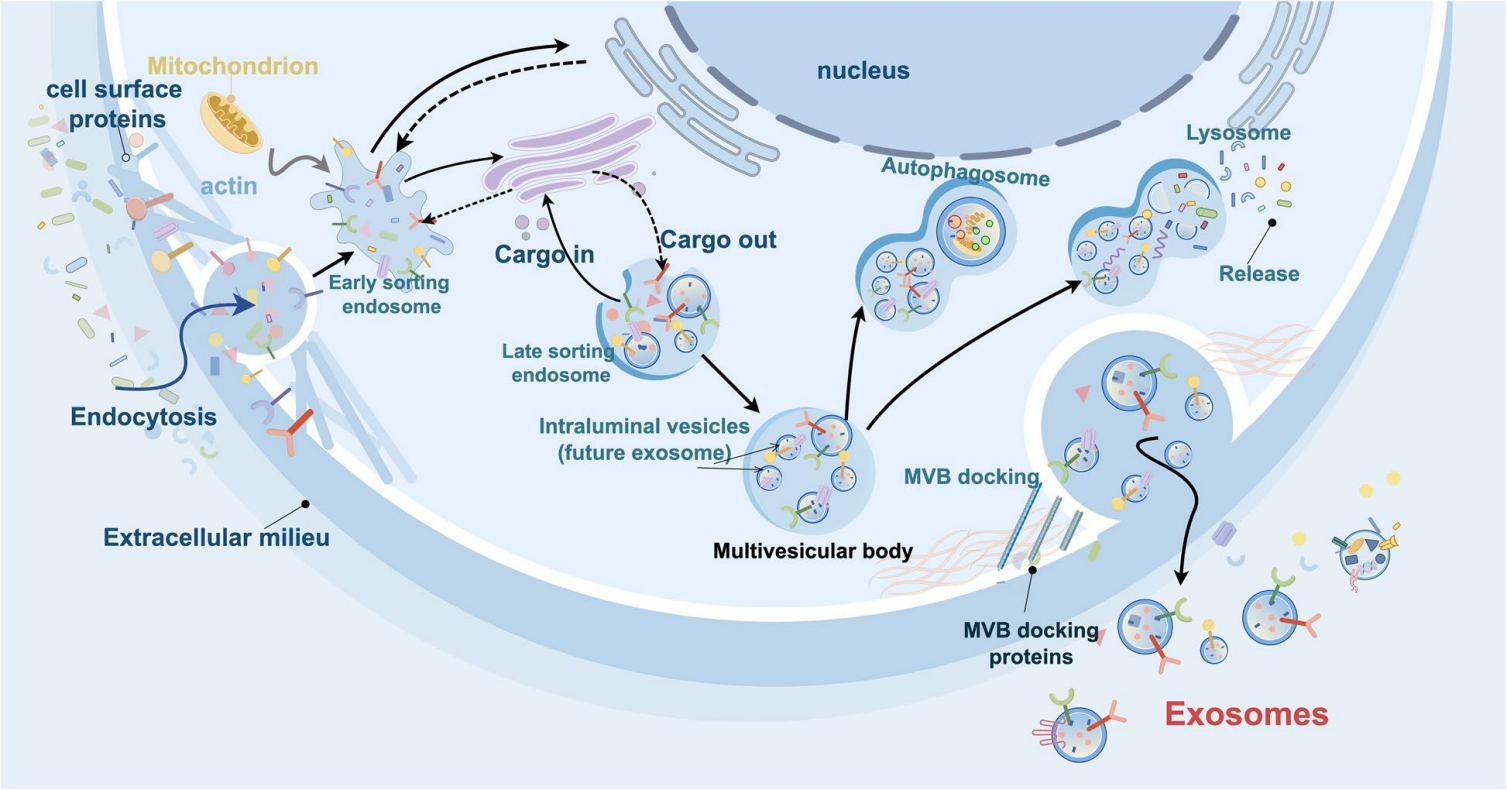
The biological roles of CDEs in gastrointestinal cancer

Exosomal biogenesis involves several steps: ①Facilitated by Golgi complex, the plasma membrane undergoes invagination to form the cup-shaped structure called early-sorting endosome (ESE) which contains the receptors, proteins, and others. ②ESEs maturation and conversion to late-sorting endosomes (LSEs). Both ESEs and LSEs can exchange substances with Golgi and Nucleus. ③LSEs then generate multivesicular bodies (MVBs), which are formed by endosomal limiting membrane inward invagination. Because of this process, MVBs contain several intraluminal vesicles (ILVs), the future exosomes. ④MVBs combine with MVB docking proteins to release exosomes; otherwise, the MVBs will degrade if they fuse with lysosomes or autophagosomes [8, 21]. This process whether the exosome will be released or degraded by lysosomes or autophagosomes, is determined by the endosomal sorting complexes required for transport (ESCRT) which contribute to exosome biogenesis [22] (Fig. 2).

**Fig. 3:**
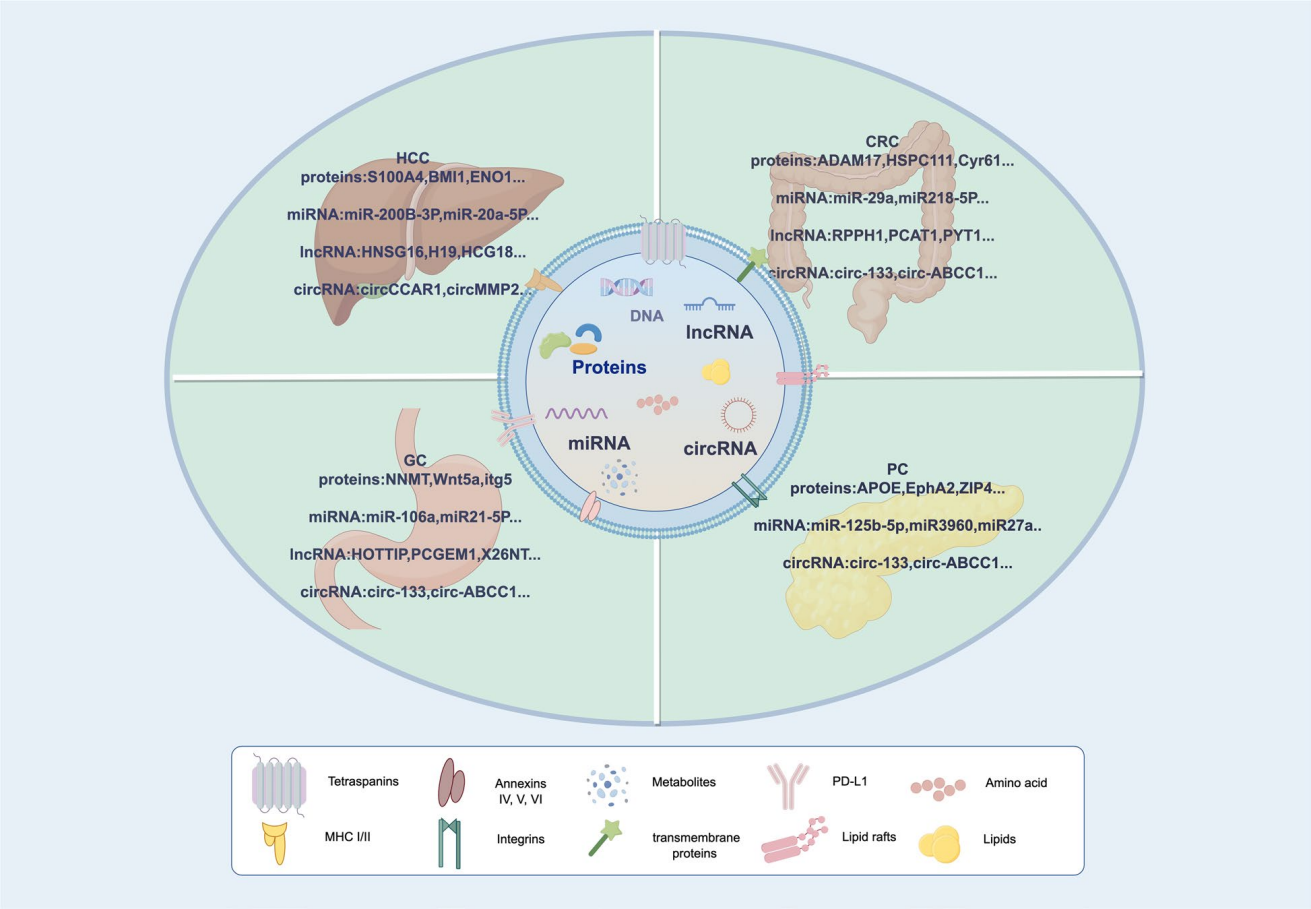
The hallmarks of CDEs in gastrointestinal cancer

Theoretically, exosomes are produced by the majority of cells [23], but the quantity of CDEs significantly surpasses that of healthy cells [12]. Riches A et al. demonstrated that within 24 h, compared with exosomes released from normal cells ((4.5 ± 2.3) × 108 exosomes per 106 cells), exosomes secreted by cancer cell lines are (53.2 ± 1.6) × 108 per 106 cells, which are obviously higher than normal cells derived exosomes [24]. Moreover, the content of exosomes is highly variable between cancer and normal tissue sources, and depending on cell origin, exosome cargo from cancer cells can be altered [8, 12, 25, 26]. Melo et al. found that exosomes derived from breast cancer cells contain massive microRNAs (miRNAs) which are way more than exosomes released from normal cells [26]. Meanwhile, in different cell types, exosomes might have diverse functions. For instance, some exosomes can interact with host immune cells, including macrophages, B cells, and T cells, to promote cancer metastasis, immune invasion, and transfer antigens to dendritic cells [27, 28]. The bioactive molecules in exosomes mirror the pathological state of the cells and tissues they obtained from, reflecting the composition of the donor cell [23, 29]. Exosomes contain a complex of diverse proteins, including receptors, transcription factors, enzymes, GTPases, annexins, cell-surface proteins, cytosolic proteins, ANNEXIN, RAB proteins, ITGs, tetraspanins (CD9, CD81, CD63, and CD82), heat shock proteins (HSP90, HSP70), and MHC proteins; DNA; different RNAs, including miRNAs, circular RNAs (circRNA), long non-coding RNAs (lncRNAs), as well as ribosomal RNAs; lipids; and metabolites [22, 25, 27, 30, 31] (Fig. 3).

### Pre-metastatic niche

The pre-metastatic niche (PMN) refers to the microenvironment orchestrated by the primary tumor in distant metastases which foster a conducive milieu to the colonization of tumor cells. Research has elucidated that the prerequisite for PMN initiation is that exosome wrap the bioactive substances released by primary tumor cells, breach the tumor invasion barriers, and directly transported through the blood circulation to reach the site of metastasis, thereby instigating local PMN formation [32]. PMN plays a pivotal role in contributing to metastatic colonization, and mainly includes six components: immunosuppression, vascular permeability/angiogenesis, organotropism, lymphangiogenesis, inflammation, and reprogramming [33]. Within this context, the immunosuppression at the metastatic site is regarded as a pivotal initiation step for the establishment of PMN, which primarily formation through ①intricate interplay of CDEs interacting with stromal cells. ②recruit, silence immune cells, downregulate the expression of immune cells surface antibody, and inhibit immune cell activation. CDEs block the T cell signaling cascade to restrain T cell activation [32, 34]. ③CDEs recruit vascular endothelial cell to promote PMN formation to enhanced cancer proliferation and metastasis [35]. ④reprogram ECM. CDEs can carry bioactive factors to promote ECM remodeling [14]. ⑤infiltrate tumor cells (Fig. 1) [19]. Overwhelming evidence shows that exosomes and their contents, especially from highly malignant tumor optimize the pre-metastatic microenvironment for GIC colonization, outgrowth, and migration by helping the metastatic cells to escape immune surveillance, transporting inflammatory factors, and increasing vascular permeability [36–38]. Recently, Shao Y et al. demonstrated that exosomal miR-21 can specifically deliver to the liver and facilitate organ-specific, distant metastasis by establishing an inflammatory PMN [39]. Meanwhile, Bruno et al. reported that the migration inhibitory factor (MIF) is highly expressive in pancreatic ductal adenocarcinoma (PDAC) derived exosomes which will be uptake by liver Kupffer cells (KCs) to release transforming growth factor beta (TGFβ) to activate fibrotic pathways to promote liver PMN formation and metastasis [38]. Zhou et al. reported that miR-105-containing exosomes efficiently destroy the tight junctions and integrity of endothelial monolayers to reach ideal organs to increase vascular permeability, thereby facilitating metastasis [40].

### Cancer-associated fibroblasts

It is clear that cancer-associated fibroblasts (CAFs), an integral part of tumor microenvironment (TME), are vital contributors to cancer progression and metastasis [41]. Not only do the CAFs provide physical support for epithelial cells, but they also secrete a variety of cell growth factors, inflammatory ligands, numerous chemokines and cytokines, and ECM proteins, such as HGF, CXCL12, insulin-like growth factor (IGF), epidermal growth factor (EGF), IL-8, IL-6, and IL-11, which are carried by exosome to influence EMT; interact with cancer cells to increase cancer proliferation, migration, invasion; induce immunosuppression and chemotherapy resistance; and remold ECM [42]. Wang et al. found that CAFs tend to uptake miR-146a-5p and miR-155-5p via exosomes to promote CRC metastasis via activating NF-κB/JAK2–STAT3 signaling to upregulate the expression of inflammatory cytokines including tumor necrosis factor-α, interleukin-6, CXCL12, and TFG-β which trigger EMT and facilitate CRC distant metastasis [43]. Recently, a study demonstrates that CAF-derived exosomes containing miR-20a-5p exert functions on regulating the TME of HCC to facilitate HCC metastasis, and EMT procession by targeting the Wnt/β-Catenin signaling pathway [44].

### Epithelial-mesenchymal transition

EMT is a biological process that can significantly enhance the ability of cell migration, which means cells lose the characteristics of epithelial cell polarity and intercellular adhesion capacity and transform into mesenchymal phenotype cells with high migration and invasion ability. EMT can implicate both physiological (including embryogenesis, wound healing, tissue regeneration, and fibrosis) and pathological processes (including cancer cell proliferation, invasion, migration, and metastasis) [45]. During the cancer microenvironment, with the alteration of ECM by CAFs, exosomes carry a large cohort of cytokines and chemokines to influence the cell–cell interaction to promote tumor progression and metastasis [46]. Zhang et al. have demonstrated that exosomal circNRIP1 can be propagated by exosomal communication between GC cells and promote cancer proliferation, migration, invasion, and metastasis by activating AKT/mTOR axis which imposes a positive impact on EMT [47]. Of note, a recent study has reported that HCC can enhance cancer proliferation and migration by secreting exosomal circ-0004277 to inhibit ZO-1 and stimulate EMT of peripheral cells through intercellular communication [48].

### Integrins

ITGs, which are the large cell adhesion receptor family and contain α and β two transmembrane glycoproteins, relate to the regulation of ECM to interact with ECM proteins and participate in cell adhesion procession. Consolidated evidence has demonstrated that CDEs express particular ITGs, including α1β1, α2, α2β1, αvβ3, αvβ5, αvβ6, α4, α4β1, α5β1, α6β1, α6β4, and α9β1, which can address CDEs into specific organs and target cells in a tissue-specific fashion and interact with ECM proteins to initiate PMNs formation and promote cancer cell proliferation, invasion, migration, and metastasis [49]. Functionally, ITGs educate cancer metastasis with two novel cooperative mechanisms: selection of target tissues to form new tumor niches during metastatic spread by ITGs carried on the exosome’s surface and horizontal transfer of integrin transcripts as vesicle cargo [18, 49, 50].

Plenty of studies have highlighted that exosomal ITGs play a crucial role in cancer progression by directly fusing with receptors in tissue-specific manner, thus proposing PMN formation and mediating organ-specific colonization [18]. Of note, exosomal ITGs α5β1, α2, αvβ3, and αvβ5 were linked to liver metastasis by modifying the local microenvironment. Oliver et al. suggested that inhibiting the expression of integrin α5β1 can significantly decrease tumor cell proliferation and reduce liver metastasis formation by suppressing the activation of endothelial cells [51]. Yoshimura et al. analyzed the primary CRC patients coexisting liver and lung metastasis and revealed that ITGs α2 expression leads to liver metastases by binding to collagen type IV [52].

### Immunosuppression

Immune system is the most powerful barrier to cancer metastasis. Massive studies have demonstrated that, within the tumor local inflammatory microenvironment, CDEs can transport proteins similar to parent tumor cells, proinflammatory chemokines, and cytokines, such as IL-35, IL10, TNF-α, IFN-γ, and TGF-β2, genomic DNA, mRNA, and microRNAs to interact with immune cells, including B cells, T cells, natural killer (NK) cells, regulatory T cells (Tregs), and tumor-associated macrophages (TAMs), thus weakening antitumor immune responses and escaping from immune surveillance [53, 54]. CDEs can ①influence immune cell maturity: impair DC maturation by influencing IL-6 expression [55], ②inhibit immune cell proliferation: impact T cell proliferation by targeting TGF-β and NK cells proliferation and cytotoxic functions via downregulating NK associated proteins [56, 57], ③inhibit the functions necessary for antitumor responses, ④induce apoptosis of activated immune cells, ⑤suppress immune cells activity, ⑥interfere with monocyte differentiation, ⑦skew the differentiation of myeloid precursor cells, ⑧polarize immune cells into tumor-promoting phenotypes [53]. Extracellular circRNAs can mediate the interaction between GIC cells and immune cells including neutrophils, NK cells, and TAM [29]. Wang et al. reported that downregulating HCC cell-derived exosomal has_circ_0074854 can inhibit HCC invasion and migration by inhibiting M2 polarization of macrophages [58]. Chen et al. reported that exosomes pack immunosuppressive proteins such as program death ligand 1 (PD-L1) on the surface to suppress CD8 + T cells function and facilitate cancer progress [59]. HCC cell-derived exosomal circTMEM181 contributes to immunosuppressive microenvironment formation, upregulates CD39 expression in macrophages, and is resistant to anti-programmed cell death 1 (PD1) treatment in HCC [60].

## Exosomes as prognostic, diagnostic and predictive biomarkers for GIC metastases

Constantly, the gold standard for the diagnosis of GIC and metastasis is image assessment combined with tissue biopsy. To date, for CRC and GC identification the gold standard is colonoscopic tissue biopsy under anesthesia [61]. In the case of HCC and PC, the established gold standard is CT-guided puncture biopsy. Elevated malignancy and the intrinsic tumor heterogeneity constitute the principal determinants underlying GIC recurrence. However, in order to surveillance of disease advancement, iterative biopsies are imperative, so patient compliance may decrease owing to the elevated risk linked to recurrent traumas and medical implantation. For patients who incapable of undergoing anesthesia and confronted with challenges in procuring biopsies from the ileocecum, duodenum, cephalic" and "caudal" regions of the pancreas, and difficult-to-biopsy patients, the feasibility of real-time monitoring of GIC progression is substantially diminished. The traditional diagnostic paradigm is undergoing transformation with the growing recognition of exosomes as indispensable elements in the diagnostic arsenal, furnishing a nuanced and comprehensive comprehension of metastases in GIC. In contrast, the swift progress in cancer examination has underscored limitations in the conventional approach of tissue biopsy. This invasive method, susceptible to advanced metastasis, confines its utility to a singular snapshot of cancer information, thereby inadequately capturing the intricate heterogeneity within the TME. On the opposite, exosomes exhibit notable advantages and stamp significant superiority over other liquid biopsy sources due to ① Consistent with the content mentioned earlier, CDEs transport a substantial protein, non-coding RNA, and DNA, facilitating intercellular communication and fostering cancer progression (Fig. 3) [22]. ② Exosomes exhibit extensive distribution within a variety of bodily fluids, thereby augmenting the practicality of their application in clinical disease assessments [23, 62]. ③ Exosomes possess high stability encapsulated by phospholipid bilayer membrane, which allows the researcher to store for a long time. ④ The approaches to get exosomes are simple, noninvasive, and real-time, so regular examinations do not cause a significant burden for patients, allowing for precise assessment of cancer progression and relapse [63, 64]. ⑤Exosomes show the potential for multicomponent analysis. ⑥Exosomes reflect the proteome and bio-information of the originating cell. ⑦Exosomes can present surface-specific proteins from parental cells or even target cells. ⑧Exosomes show superior performance over traditional serum-based biomarkers [25, 62]. As previously elucidated, established evidence underscores that GIC derived exosomes assume a critical role in mediating CRC, PC, HCC, and GC distant metastasis, especially invasive involvement with the liver, lungs, and brain. CDEs circular in biofluids and carry diverse GIC-specific bioactive constituents, (encompassing proteins, miRNA, circRNA, lncRNA, and active factor) to enhance the migratory capacity of GIC cells [65], assistant PMN and CAF formation which creates a hotbed for GIC colonization [39], cross-talking with immune cells, and destroy vascular endothelial barrier to advance vascular permeability [66]. Table 1 furnishes a comprehensive summary of different exosomal proteins, miRNAs, circRNAs, and lncRNAs between patients with CRC, HCC, GC, and PC serving as the minimally invasive and conspicuous heterogeneity source of potential diagnostic, prognostic, and predictive biomarkers.

**Table 1:** Potential exosomal biomarkers in GIC metastasis and their mechanism

Cancer type	Exosomal Contents	Exosome Source	Exosomal Cargo	Effect	Clinical Significance	Ref
colorectal cancer	miRNA	Cell culture fluid	miR-29a	downregulate the expression of ZO-1, Claudin-5, and Occludin via targeting KLF4	promote metastasis	[66]
		Cell culture fluid	miR-218-5p	activate the Ras/ERK/c-Fos pathway	promote metastasis	[110]
		Cell culture fluid	miR-181b-3p	downregulate SNX2 expression	promote metastasis	[111]
		serum	miR-92a-3p	inhibit FBXW7 and MOAP1	promote metastasis	[104]
		Cell culture fluid	miR-27b-3p	activate STAT3 pathway	promote metastasis	[113]
		Cell culture fluid	miR-203a-3p	inhibit the Src/PKC/GSK-3β	promote metastasis	[108]
		Serum	miRNA132-5p, miRNA6087, and miRNA320d	not mention	promote metastasis	[189]
		Serum	miR-146a-5p and miR-155-5p	activate JAK2–STAT3/NF-κB signaling	promote metastasis	[43]
		Cell culture fluid	miRNA-335-5p	overexpress of RASA1	promote metastasis	[190]
		Serum	miR-122	not mention	promote metastasis	[191]
		Serum	miR-141-3p and miR-375	not mention	promote metastasis	[91]
		Serum	miR-106b-3p	downregulate DLC-1 expression	promote metastasis	[92]
		Serum	miR-1246/92b-3p/27a-3p	target GSK3β and activate the Wnt/β-catenin pathway	promote metastasis	[65]
		Serum	miR-193a and let-7 g	not mention	promote metastasis	[192]
		Serum	miR-128-3p	activate TGF-β/SMAD and JAK/STAT3 signal pathway	promote metastasis	[102]
		Cell culture fluid	miR-106b-5p	downregulate PDCD4 and activate the PI3Kγ/AKT/mTOR signaling pathway	promote metastasis	[193]
		Cell culture fluid	miR-21-5p	suppress VHL/ HIF-1α axis, target TrkA/ERK/ELK1 pathway	promote metastasis	[194]
		Serum	miR-934	downregulate PTEN expression and activate the PI3K/AKT signaling pathway	promote metastasis	[195]
		Serum	miR-25-3p, miR-130b-3p, miR-425-5p	activate of the CXCL12/CXCR4/PTEN/PI3K/Akt pathway axis	promote metastasis	[196]
		Serum	miR- 221/222	suppress SPINT1 expression	promote metastasis	[94]
		Serum	miR-1229	inhibit the expression of HIPK2, activate VEGF pathway	promote metastasis	[197]
		Cell culture fluid	miR-183-5p	suppress FOXO1 expression	promote metastasis	[112]
	circRNA	serum	circPABPC1	upregulate HMGA2, BMP4 and ADAM19 expression	promote metastasis	[139]
		Serum	circ_FMN2	serve as miR-338-3p Sponge to downregulate MSI1	promote metastasis	[198]
		Cell culture fluid	circPACRGL	facilitate the TGF-β1 expression, regulate miR-142-3p/miR-506-3p-TGF-β1 axis	promote metastasis	[199]
		Tissue	circLONP2	not mention	promote metastasis	[140]
		Cell culture fluid	circ-ABCC1	activate the Wnt/β-catenin pathway	promote metastasis	[200]
		serum	circ-133	activate the miR-133a/GEF-H1/RhoA axis	promote metastasis	[136]
		serum	circGAPVD1	not mention	promote metastasis	[142]
		serum	circTUBGCP4	activate Akt signaling pathway, sponge with miR-146b-3p	promote metastasis	[135]
		serum	hsa-circ-0004771	not mention	promote metastasis	[201]
		Cell culture fluid	ciRS-122	ciRS-122-miR-122-PKM2 pathway	promote metastasis	[151]
		serum	circCOG2	Activate the miR-1305/TGF-β2/SMAD3 pathway	promote metastasis	[202]
		Cell culture fluid	circLPAR1	suppress the METTL3-eIF3h interaction, decrease the translation of oncogene BRD4	promote metastasis	[203]
	lncRNA	Cell culture fluid	lnc-HOXB8-1:2	bind hsa-miR-6825-5p to upregulate CXCR3 expression	promote metastasis	[204]
		Cell culture fluid	lncRNA RPPH1	interact with protein TUBB3	promote metastasis	[122]
		serum	lncRNA PVT1/VEGFA	downregulate miR-152-3p	promote metastasis	[120]
		Cell culture fluid	lncRNA PCAT1	activate of the miR-329-3p/Netrin-1-CD146 Complex	promote metastasis	[127]
		Cell culture fluid	lncRNA BANCR	regulate RhoA/ROCK signaling	promote metastasis	[205]
		Cell culture fluid	lncRNA MALAT1	sponge miR-26a/26b and activate PI3K/Akt/mTOR pathway	promote metastasis	[128]
		Cell culture fluid	lncRNA UCA1	sponge with miR-143	promote metastasis	[117]
	proteins	serum	ADAM17	cleaving the E-cadherin junction	promote metastasis	[69]
		serum	HSPC111	activate CXCL5-CXCR2 axis	promote metastasis	[68]
		serum	KRAS	not mention	promote metastasis	[71]
		serum	Cyr61	activate αV β5 /FAK/HIF-1α/STAT3/MMP2 signaling	promote metastasis	[83]
		Cell culture fluid	FMNL2	activate the EGFL6/CKAP4/ERK axis	promote metastasis	[84]
		Cell culture fluid	ADAM17	not mention	promote metastasis	[70]
hepatocellular cancer	miRNA	serum	miR-200b-3p	activate JAK/STAT signaling pathway	promote metastasis	[95]
		liver tissue	miR-20a-5p	downregulate LIMA1-Mediated β-Catenin Pathway	promote metastasis	[44]
		Cell culture fluid	miR-412,36 miR-4286,37 miR-423–5p, and miR-29a-5p,	impair lysosome biogenesis	promote metastasis	[206]
		Cell culture fluid	miR-452-5p	downregulate TIMP3	promote metastasis	[207]
		serum	miR92a-3	inhibit PTEN and activating Akt/Snail signaling	promote metastasis	[107]
		Cell culture fluid	miR-21 and miR-10b	not mention	promote metastasis	[208]
		serum	miR-18a, miR-20b, and miR-221	not mention	promote metastasis	[96]
		serum	miR-1307-5p	downregulate miR-1307-5p/SEC14L2/Akt and miR-1307-5p/ENG signaling pathways	promote metastasis	[97]
		Cell culture fluid	miR-1273f	activate the Wnt/β-catenin signaling, and downregulate LHX6	promote metastasis	[209]
		Cell culture fluid	miR-100-5p	downregulate CLDN11, activate PI3K/AKT signaling pathway	promote metastasis	[210]
		serum	miR-4661-5p	not mention	promote metastasis	[101]
	circRNA	Cell culture fluid	circ_002136	downregulate miR-19a-3p/RAB1A pathway	promote metastasis	[211]
		serum	circCCAR1	upregulate WTAP	promote metastasis	[149]
		Cell culture fluid	circ_0003028	activate miR-498/ODC1 signaling axis, sponge miR-498,	promote metastasis	[212]
		Cell culture fluid	circ_MMP2	upregulate of its host gene MMP2 via sponging of miR-136-5p	promote metastasis	[213]
		HCC tissues	circTTLL5	activate miR-136-5p/KIAA1522 axis, sponge miR-136-5p	promote metastasis	[137]
		serum	circRNA-100338	activate of MMP9, regulate VM formation	promote metastasis	[214]
		serum	circGSE1	activate the miR‐324‐5p/TGFBR1/Smad3 axis	promote metastasis	[215]
		serum	CircANTXR1	activate miR-532-5p/XRCC5 axis	promote metastasis	[216]
		HCC tissues	CircPAK1	bind 14–3–3 ζ with YAP	promote metastasis	[217]
		Cell culture fluid	circPTGR1	activate miR449a-MET pathway	promote metastasis	[141]
		Cell culture fluid	Circ-0000284	not mention	promote metastasis	[134]
		plasma	circUHRF1	inhibit NK cell function	promote metastasis	[150]
		Plasma	hsa_circ_0004277	inhibit of ZO-1	promote metastasis	[48]
		Cell culture fluid	hsa_circ_0074854	mediate macrophage M2 polarization	promote metastasis	[58]
		Cell culture fluid	CircRNA Cdr1as	promote the expression of AFP and sponge with miR-1270	promote metastasis	[218]
		Cell culture fluid	circTMEM181	inhibit the ATP-adenosine pathway, increase the expression of CD39	promote metastasis	[60]
		serum	Circ-ZNF652	vie miR-29a-3p/guanylyl cyclase domain containing 1 axis	promote metastasis	[219]
	lncRNA	serum	LINC00161	inhibiting miR-590-3p to activate the ROCK2 signaling pathway	promote metastasis	[119]
		serum	lncRNA THEMIS2- 211	activate THEMIS2-211/miR-940/SPOCK1 axis	promote metastasis	[121]
		serum	lncRNA SNHG16	activate miR-942-3p/MMP9 axis	promote metastasis	[124]
		Cell culture fluid	lncRNA H19	activate miR-520a-3p/LIMK1 axis	promote metastasis	[220]
		Cell culture fluid	lncRNA HCG18	downregulate miR-424-5p/SOX9 axis and PI3K/AKT pathway	promote metastasis	[221]
		serum	lncRNAENSG00000248932.1, ENST00000440688.1 and ENST00000457302.2	not mention	promote metastasis	[222]
	proteins	Cell culture fluid	CTLA-4	activate PTEN/CD44 signal pathway	promote metastasis	[85]
		Cell culture fluid	S100A4	activate STAT3 phosphorylation and up-regulating OPN expression	promote metastasis	[74]
		Cell culture fluid	BMI1	not mention	promote metastasis	[79]
		tumor tissue	LOXL4	activate the FAK/Src pathway	promote metastasis	[82]
		Cell culture fluid	ENO1	activate the FAK/Src-p38MAPK pathway	promote metastasis	[81]
		Cell culture fluid	RAB13	upregulate VEGF and activity of CUX1	promote metastasis	[223]
		Cell culture fluid	RAB5A	not mention	promote metastasis	[224]
gastric cancer	miRNA	peritoneal lavage	hsa-let-7 g-3p and hsa-miR-10395-3p	activate the TGFβ signaling pathway	promote metastasis	[225]
		Cell culture fluid	MiR-374a-5p	activate NF-κB signaling	promote metastasis	[226]
		serum	miR-519a-3p	activate the MAPK/ERK pathway by targeting DUSP2	promote metastasis	[98]
		Cell culture fluid	miR-106a	inhibit the expression of Smad7	promote metastasis	[227, 228]
		Cell culture fluid	miR-21-5p	activate TGF-β/Smad pathway	promote metastasis	[229]
		serum	miR-10b-5p, miR-101-3p and miR143-5p	not mention	promote metastasis	[114]
		serum	miR-301a-3p	activate MiR-301a-3p/PHD3/HIF-1α signaling axis	promote metastasis	[230]
		Cell culture fluid	miR-501-5p	downregulate of BLID, subsequent inactivate of caspase-9/-3 and phosphorylate of Akt	promote metastasis	[231]
		serum	miR-379-5p and miR-410-3p	not mention	promote metastasis	[99]
		Cell culture fluid	miR-486-5p	downregulate SMAD2, CDK4, and ACTR3	promote metastasis	[232]
		serum	hsa‐miR‐148‐3p, hsa‐miR‐335‐5p, hsa‐miR‐3613‐5p, and hsa‐miR‐556‐5p	not mention	promote metastasis	[233]
		Peritoneal lavage fluid	miR-21-5p, miR-92a-3p, miR-223-3p, and miR-342-3p	not mention	promote metastasis	[106]
		serum	miR-196a-1	inhibit SFRP1 protein expression	promote metastasis	[234]
		serum	miR-1307-3p、piR-019308、piR-004918, piR-018569	not mention	promote metastasis	[100]
	circRNA	serum	circ_0038138	downregulate miR-198/EZH2 axis	promote metastasis	[235]
		serum	circFCHO2	activate the JAK1/STAT3 pathway via sponging miR-194-5p	promote metastasis	[236]
		Tumour tissues	circNRIP1	activate AKT1/mTOR pathway, sponge of miR-149-5p	promote metastasis	[47]
		serum	circNEK9	activate miR-409-3p/MAP7 axis	promote metastasis	[133]
		serum	Hsa_circ_0000437	upregulate SRSF3, activate HSPA2-ERK signaling pathway	promote metastasis	[138]
		serum	circ-RanGAP1	activate miR-877-3p/VEGFA axis	promote metastasis	[143]
		serum	circSHKBP1	activate the miR-582-3p/HUR/VEGF pathway	promote metastasis	[144]
		Cell culture fluid	circular RNA UBE2Q2	via the circUBE2Q2-miR-370-3p-STAT3 axis	promote metastasis	[237]
		Cell culture fluid	circular RNA circ_0032821	sponge with miR-515-5p to regulate SOX9 expression	promote metastasis	[238]
		serum	circ-PVT1	by miR-30a-5p/YAP1 axis	promote metastasis	[239]
	lncRNA	Cell culture fluid	lncRNA HOTTIP	activate microRNA-885-3p/EphB2 axis	promote metastasis	[240]
		serum	lnc-SLC2A12-10:1	not mention	promote metastasis	[241]
		Cell culture fluid	lncRNA PCGEM1	reduce the degradation of SNAI1	promote metastasis	[126]
		tumor tissue	lncRNA SPRY4-IT1	activate SPRY4-IT1/miR-101-3p/AMPK axis	promote metastasis	[118]
		Cell culture fluid	lncRNA TTN-AS1	activate miR-499a-5p/ZEB1/CDX2 axis	promote metastasis	[125]
		tumor tissue	lncRNA LINC01091	activate miR-128-3p/ELF4/CDX2 axis	promote metastasis	[109]
		serum	lncRNA X26nt	decrease vascular endothelial cadherin	promote metastasis	[242]
	proteins	peritoneal lavage fluid	NNMT	activate TGF-β/smad2 signal pathway	promote metastasis	[75]
		serum	Wnt5a	inhibit of YAP signal pathway	promote metastasis	[72]
		serum	IntegrinB5	activate PI3K-AKT pathways	promote metastasis	[73]
pancreatic cancer	miRNA	Cell culture fluid	miR-125b-5p	activate of MEK2/ERK2 signaling	promote metastasis	[243]
		serum	miR-3960	suppress TFAP2A/PTEN/AKT signaling pathway	promote metastasis	[244]
		Cell culture fluid	miR-27a	induce angiogenesis by inhibiting BTG2 expression	promote metastasis	[245]
		Cell culture fluid	miR-501-3p	decrease TGFBR3 levels and activate TGF-βpathway	promote metastasis	[246]
	circRNA	serum	circ-IARS	sponge with miR-122	promote metastasis	[145]
		Cell culture fluid	circ-PED8A	upregulate MET, sponge with miR-338, activate MACC/MET/ERK or AKT pathways	promote metastasis	[146]
		serum	circZNF91	MiRNA sponge for miR-23b-3p	promote metastasis	[152]
	proteins	serum	EphA2	not mention	promote metastasis	[247]
		Cell culture fluid	CD44v6/C1QBP complex	activate of IGF-1 signal	promote metastasis	[76]
		serum	APOE	not mention	promote metastasis	[248]
		Cell culture fluid	Eps8	not mention	promote metastasis	[78]
		Cell culture fluid	ASPH	activate Notch signaling	promote metastasis	[86]
		serum	DNAJB11	activate the EGFR/MAPK pathway	promote metastasis	[87]
		serum	CCT8, CTSL, SAA1, IGF2	not mention	promote metastasis	[88]
		cancer tissue	FGD5-AS1	activate STAT3/NF-κB signaling pathway	promote metastasis	[89]
		serum	Lin28B	activate the Lin28B/let-7/HMGA2/PDGFB signaling pathway	promote metastasis	[90]
		Cell culture fluid	ZIP4	not mention	promote metastasis	[249]
		serum	PRKD-1	increase in F-actin	promote metastasis	[250]
		Cell culture fluid	DYRK1A	stabilize the c-MET receptor through SPRY2, lead to prolonged activate of extracellular signal-regulated kinase signaling	promote metastasis	[77]

*Abbreviations*: *TGF-β1* transforming growth factor-β1, *VM* vasculogenic mimicry, *VEGF* vascular endothelial-derived growth factor, *IGF-1* insulin-like growth factor 1, *DYRK1A* Dual-specificity tyrosine regulated kinase 1A, *SPRY2* Sprouty 2, *HUR* human antigen R

### Exosomal proteins as the biomarkers of GIC

Some investigations scrutinizing the biomarkers associated with GIC metastasis have predominantly centered their attention on the impact of exosomal proteins. Conducting proteomic assays and juxtaposing biofluids-derived exosomal proteins from cancer-afflicted individuals with those from cancer-free counterparts reveals substantial disparities in both the quantity and composition of exosomal proteins. Moreover, exosomal proteins sourced from diverse origins and subject to distinct conditions exhibit marked heterogeneity [67]. Upon co-culturing those highly heterogeneous exosomes with tumor cells and nude mice, it was found that exosomal proteins exert a stimulatory influence on the migratory capability of cancer cells, augment the metastatic nodules and activate several pathways usually involved in cancer development. Hence, exosomal proteins serve as dependable prognostic, diagnostic, and predictive biomarkers for the detection of GIC recurrence.

Various investigators have identified that, relative to healthy peoples or non-metastatic patients, five exosomal proteins (exosomal HSPC111, ADAM17, KRAS, Wnt5a, and IntegrinB5) are significantly overexpressed in patients afflicted with highly metastatic CRC and GC [68–73]. And these proteins are downregulating after lumpectomy, escalating the formation of metastatic lesions after injecting into nude mice, and triggering respective downstream signal pathway to facilitate cancer metastasis. The collective observations substantiate that exosomes directly sourced from tumors cells, harboring neoplastic information, thereby implying that those proteins serving as potential diagnostic biomarkers for GIC. In addition to serum, exosomal levels of S100A4, NNMT, CD44v6/C1QBP, DYRK1A, BMI1, and Eps8 derived from tumor tissues are also elevated in highly-metastatic HCC, GC, and PC patients [74–79]. And those exosomal proteins actively participate the aforementioned tumor pre-metastatic process to advance cancer recurrence.

Indeed, the abundance of numerous exosomal proteins manifests as a dynamic and multifaceted profile rather than a static, unidimensional state. This dynamic nature is characterized by elevation across a broad spectrum of metastatic GIC, serving as a diagnostic marker for diverse cancers. For instance, exosomal S100A4 exhibits heightened levels in both metastatic HCC and PC [74, 80], whereas IntegrinB5 is a diagnostic indicator in GC but a prognostic indicator in PC [73, 80]. Li and jiang et al. discerned that the expression of exosomal LOXL4, and ENO1 is also been identified to be overexpressed in HCC patients and intimately associated with the tumor-node-metastasis (TNM) stage, and the protein within serum-derived exosomes is intricately linked to an unfavorable prognosis in HCC patients [81, 82]. Liang et al. found that plasma exosomal Cyr61 was significantly higher in TNM stages III and IV (*n* = 185) compared with stages I and II (*n* = 179). Furthermore, in contrast to traditional diagnostic marker carcinoembryonic antigen (CEA) and carbohydrate antigen (CA19-9), serum Cyr61 exhibit superior diagnostic and prognostic efficacy for CRC according to multivariate logistic regression analysis and receiver operating characteristic (ROC) area under the ROC curve (AUC) of 0.933 [83]. Exosomal proteins, with the ability to discern between the early and advanced stages of GIC, emerge as commendable indicators to guide cancer treatment.

Other exosomal proteins have also been identified as important predictive biomarkers of GIC owing to create cancer-friendly condition to promote cancer metastasis, like exosomal Formin-like 2 (FMNL2), cytotoxic T lymphocyte antigen 4 (CTLA-4), RAB5A, aspartate β-hydroxylase, and so on [84–90]. While direct evidence linking them to TNM stage and serous content remains elusive, these exosomal proteins exhibit the capacity to orchestrate conditions and stimulate signaling pathways that facilitate the conversion of the internal environment in cancer patients, making it prone to metastasis, thereby assuming the responsibility of predictive biomarkers for GIC. For instance, CRC FMNL2 overexpression can promote CRC angiogenesis and metastasis by activating the ERK/MMP signal pathway [84], exosomal CTLA-4 could mediate the proliferation, invasion, and metastasis of HCC cells by mediating PTEN/CD44 signal pathway [85], and exosomal DNAJB11 can activate the downstream MAPK signaling pathway, which would enhance PC invasive ability [87, 88]. Another study demonstrated that exosome-derived FGD5-AS1 can activate STAT3/NF-κB pathway to mediate M2 macrophage polarization and advance the malignant behaviors of PC [89].

### Exosomal miRNA as the biomarkers of GIC

Proteins represent merely a facet of exosomal carryover and may exhibit limitations as biomarkers for GIC recurrence. Consequently, a multitude of studies have shifted their attention towards on the impact of exosomal miRNA on the induction of cancer progression, which constitutes as the most prevalent constituent within exosomes. Melo et al. found that comparing with normosomes, exosomal miRNAs originating from cancerous sources manifest a surge of up to a 30-fold increment [26]. Other researchers found that some miRNAs, including miR-29a, miR-141-3p, miR-375, miR-106b-3p, miR- 221/222, miR-200b-3p, miR-18a, miR-20b, miR-1307-5p, miR-519a-3p, miR-379-5p, and miR-410-3p are also overexpression in metastatic CRC, HCC, and GC patients. The expression of these miRNAs notably diminishes post-surgical intervention, substantiating their predominant derivation from GIC cells and signifying their role as predictive biomarkers of GIC [66, 91–100]. Tian et al. demonstrated that injection the normal as well as GIC patient serum-derived exosomes into tumor xenograft models, overexpressed miR-200b-3p, miR‐221, and miR‐222 was positively associated with the metastatic foci growth, and increase in the metastatic ratio and diameters of metastatic foci. Sun et al. found that miR-122 is overexpressed in CRC tissue, and miR-122 show discriminatory potential in distinguishing CRC patients with or without liver metastasis (LM) (AUC of 0.89 and 0.81). Moreover, akin to the observation in exosomal proteins, the integration of exosomal biomarkers with existing tumor markers demonstrates the potential to augment precision in monitoring GIC recurrence. Ge et al. revealed that four miRNAs is significantly increased in metastatic GC, and when amalgamated with conventional diagnostic marker CEA and CA-199, the augmentation of the diagnostic accuracy of miR-1307-3p, piR-019308, piR-004918, and piR-018569 is apparent (the AUC separately upregulate from 0.845, 0.820, 0.754 and 0.732 to 0.902, 0.914, 0.859, and 0.868) [100]. Cho et al. elucidated that serval miRNAs are evidently elevated in early‐stage HCC (single tumor < 2 cm), miR‐4661‐5p could diagnose HCC in all stages (AUC = 0.917). The diagnostic accuracy of the serum exosomal miR‐4661‐5p (AUC = 0.923), miR‐1269a (AUC = 0.684), and miR‐25 (AUC = 0.812) in diagnosing early‐stage HCC show markedly superior AUR values than serum AFP (AUC = 0.541) [101]. Clinically, miR-128-3p is overexpressed in patients with later tumor stage (III–IV), and its expressivity is highly associated with perineural invasion, disease stage, and CA 19–9 content in CRC patients (*P* < 0.05) [102]. While these investigations adopt diverse perspectives on the metastatic process, collectively, they afford contemporaneous insights into the dynamic status of the neoplastic lesion.

Simply classification of RNA into distinct diagnostic and prognostic categories is actually a bit subtle, as certain multiple miRNA species have both diagnostic and prognostic significance. MiR-301a-3p not only increase in the serum of PC patients and its levels positively correlate with invasion depth and advanced TNM stage of PC [103]. Similarly, miR-92a-3p is prominently elevated not solely within the serum of high-metastatic CRC patients, but also HCC and GC. And miR-92a-3p is positively correlate with progressed TNM stage, and can mediate chemotherapy resistance, thereby contributing to an adverse prognosis in patients afflicted with GIC [104–107]. In addition to these two, exosomal miR-1229, miR-1307-5p manifest to this particular phenotype [97]. Apart from the aforementioned miRNAs, other miRNAs have been identified as relevant predictive biomarkers. With the assistant of miR-203a-3p, miR-128-3p, mi-335-5p, miR-106b-5p, miR-218-5p, miR-181b-3p, miR-183-5p, and miR-27b-3p, there is an augmentation in the migratory capability of CRC cells, concomitant with an elevated propensity for metastasis in CRC[92, 93, 102, 108–113]. Analyzing the serum derived exosomal miRNAs, Zhang et al. discovered that the expression profiles of miR-10b-5p, miR 143-5p, and miR 101-3p showed statistically significant up-regulation in individuals diagnosed with GC and concurrent lymph node, liver, and ovarian metastases (*p* < 0.05, AUC = 0.8919, 0.8247, 0.8905) [114]. While the extensive heterogeneity of biomarkers necessitates additional refinement and standardization, extant evidence highlights the potential of exosomal miRNAs in evaluating metastatic GIC and prognostic outcomes.

### Exosomal lncRNA as the biomarkers of GIC

Subsequent to an in-depth investigation of miRNA, researchers contemplated the potential utility of lncRNA, another noncoding RNA subtype, as a prospective biomarker for GIC detection. lncRNA is a large class of transcripts with a length exceeding 200 bp that exert functions in a bunch of biological procedures, including transcriptional, epigenetic, and post-transcriptional levels, and initiate the progression and metastasis of cancers[115]. Substantial evidence suggests that lncRNAs could mediate each step of metastasis such as cell migration, invasion, and distant-site colonization [115]. Empirical investigations disclose the diagnostic aptitude embedded within exosomal lncRNAs, furnishing enlightening perspectives on the detection of metastatic GIC [116]. For instance, luan et al. isolate the exosomal RNA from serum of both CRC patients and normal human. Subsequent analytical assessments found that 569 lncRNAs were up-regulated, concomitant with a downregulation in 475 lncRNAs [117]. LncRNA UCA1 is overexpressed in neoplasms at advanced stages (*p* = 0.007), distant metastatic patients (*p* = 0.003), and patients afflicted with tumors > 5 cm (*p* = 0.005). Analogous observations were reported by cao et al. [118]. LncRNA SPRY4-IT1 is upregulated in GC tissue (*p* < 0.001), and silencing SPRY4-IT1 results in the attenuation of GC progression. LncRNA UCA1 and lncRNA SPRY4-IT1 have shown considerable potential as prospective diagnostic biomarker for GIC. Likewise, high levels of lncRNA PVT1/VEGFA and LINC00161 were discerned in both serum and tissue of CRC and HCC patients [119, 120]. However, LncRNA THEMIS2-211 and RPPH1 concurrently functions as both a diagnostic and prognostic. within the cohort comprising of 89 HCC patients and 60 normal controls, plasmatic exosomal LncRNA THEMIS2-211 is upregulate in HCC patients (*p* < 0.001), and the diagnostic efficiency of exosomal THEMIS2-211 experiences a notable improvement when combinate with AFP in diagnosing stage I HCC patients [121]. Additionally, the expression levels of exosomal THEMIS2-211 is way superior in advanced-stage (III, IV stages) than early-stage (I, II stages). Coincidentally, LncRNA RPPH1 is also overexpressed in non-treatment CRC patients but lower after cancer resection, and have a strong connection with advanced TNM stages and poor prognosis [122]. Exosomal RPPH1 bespeak better diagnostic value (AUC = 0.86) when contrasted with CEA and CA199 as well.

Hashemi et al. reported that lncRNA H19 can trigger chemo- and radio-resistance in cancer cells, signifying that lncRNA H19 is a unitary prognostic biomarker of tumor recurrence [123]. The presence of lncRNA SNHG16, TTN-AS1 and PCGEM1 in the tissues of cancer patients accentuates the malignant features of GIC cells, culminating in a diminished survival rate with an unfavorable prognosis. Xu et al. utilized Pearson’s correlation and univariate statistical analysis to examine a cohort of 78 cases with low or high expression of LncSNHG16. Their findings unveiled a positive correlation between heightened LncSNHG16 expression and HCC relapse within a 2-year period (*p* = 0.000) [124]. Analogous studies have also been proposed by Wang et al. Bioinformatics analysis delineated an augmented expression of lncRNA TTN-AS1 in GC is highly correlation with the poor overall survival (OS) [125]. Other lncRNAs have also been identified as important predictive biomarkers of GIC. Fang et al. identified that lncRNA PCAT1 and PCGEM1 can promote EMT, thereby enhancing the proliferation and migration ability of CRC cells [126]. When knockdown of PCAT1, the metastatic propensity notable attenuated, particularly liver metastasis [127]. Simultaneously, exosomal metastases-associated lung adenocarcinoma transcript 1 (MALAT1) can advance CRC cells with high metastatic and invasive abilities [128]. While these lncRNAs contribute to GIC metastasis in diverse mechanisms, collectively they underscore the heterogeneity inherent of primary tumor cells.

### Exosomal circRNA as the biomarkers of GIC

Amidst the surge of circRNA research, some scholarly publications express a profound curiosity regarding the comparability of circRNAs to other non-coding RNAs in their potential to promote tumor metastasis, and the possibility to serve as available biomarkers for cancer recurrence [129, 130]. With deeper investigation, researchers discerned that circRNAs are characterized by rich diversity, a stable structural framework, conserved sequences, and manifest cell- or tissue-specific expression patterns. Furthermore, circRNAs function as miRNA sponges to modulate the microRNA-mRNA regulatory axis [131, 132]. This revelation implies that circRNAs manifest a significant tumor heterogeneity and hold potential as biomarkers for metastatic-GIC. Like the aforementioned non-coding RNA, circ-0000284, circFMN2, circ-ABCC1, circTUBGCP4, circ-133, circTTLL5, circPTGR1, hsa_circ_0004277, circ_0038138, and hsa_circ_0000437 are aberrantly overexpressed in the exosomal circRNAs derived from CRC, HCC, and GC patients when compare with healthy volunteers, especially in patients with distance metastasis (M1) [48, 133–138]. Moreover, the levels of these circRNAs are dramatically descend after removing primary lumps, suggesting that neoplastic cells directly contribute to the pool of circRNAs, thereby potentially serving as diagnostic biomarkers for GIC. Mechanistic analysis has revealed that these circRNAs can sponge with diverse miRNAs to activate downstream to improve the chances of metastatic foci formation in tumor xenograft models. Similar observations are reported by Li et al. The overexpression of circPABPC1 accelerates the advancement of CRC, while the ablation of circPABPC1 attenuates CRC progression [139]. In addition to the above-mentioned non-coding RNA, a number of circRNAs also manifest the diagnostic and prognostic function in detection GIC recurrence. Not only does circLONP2 and circPTGR1 upregulate in tumor patients, but also the OS rate of patients with high expression of these circRNAs is notably unfavorable (*p* < 0.05) [140, 141]. And elevated expression of circNEK9 (*I* = 30, *p* = 0.0099) and circGAPVD1 (*n* = 78, *p* = 0.011) are correlate with augmented lump size and advanced TNM stage in GC and CRC patients [133, 142]. Lu et al. conducted an assessment of circ-RanGAP1 expression in a cohort of 97 paired GC samples, and found that circ-RanGAP1 is conspicuously upregulate in stage III than stage I-II [143]. Statistical scrutiny revealed a noteworthy correlation between heightened circ-RanGAP1 expression and large tumor size (*p* = 0.016) with an advanced clinical stage (*p* = 0.001). In a comprehensive examination of circSHKBP1 expression across 72-paired GC and normal specimens, Xie et al. found a 2.31-fold upregulation of circSHKBP1 in GC than normal (*P* < 0.05), and overexpressed circSHKBP1 is highly correlate with poor prognosis and advanced TNM stage, positioning it a potential biomarker for GC [144]. These phenotypic traits are similarly present in circ-IARS, circ-YAP, and circPDE8A [145–147].

Furthermore, few circRNA is just a mono-prognosticator for GIC. For instance, sang et al. found that the expression of circRELL1 is correlated with TNM stage and a poor survival rate, making it an ideal prognostic biomarker of GC [148]. Besides, the expression of circCCAR1 is not only positively associate with CRC grade and TNM stage, but also can induce CRC patients with the resistance to anti-PD1 therapy [149]. It is not a unique instance, but has its counterpart. Overexpression of circUHRF1 and circTMEM181 can attenuate the anti-tumor efficiency of anti-PD1 therapy, consequently diminishing OS rates [60, 150]. Apart from above-mentioned circRNA, ciRS‐122 is higher in the serum of oxaliplatin‐resistant patients (*n* = 13) than oxaliplatin‐sensitive patients (*n* = 6). Enhanced ciRS‐122 expression diminishes the oxaliplatin susceptibility both in vivo and vitro, whereas targeted suppression of it can reverse this chemoresistance [151]. likewise, overexpression of circZNF91 can induce Gemcitabine resistance in PC cells [152]. These exosomal circRNAs collectively serve as valuable indicators for guiding the prognosis of GIC. A substantial body of empirical evidence now substantiates the direct origin of exosomes from neoplastic cells, encapsulating a cargo reflective of their cellular origin. These exosomes exhibit flawless heterogeneity, offering the potential to function as evaluative entities and providing insights to assess the severity of disease and monitor the prognosis of GIC in real-time. It is precisely these research results that highlight discernment and therapeutic avenues of metastatic cancer.

## The clinic application of CDEs in gastrointestinal cancer

It is well established that CDEs have particularly promising applications as liquid biopsies, because exosomes could be released by all types of cells and widely distributed in all biological fluids, and the components of CDEs reflect the biological state of their origin. Of note, some CDEs show the possibility of mediating the malignant behaviors of cancer. Based on this theory, we can utilize the characteristics of exosomes, not only to monitor the malignant behaviors of cancer but also to target CDEs as a therapeutic approach to cure cancer.

### Exosomes can inhibit cancer biogenesis

Besides the aforementioned exosomes that can trigger cancer metastasis, substantial research demonstrates that some exosomes can also inhibit cancer progression. Given this fact, we can harness exosomes or activate relative downstream to forbid cancer metastasis and cure cancer (Table 2).

**Table 2:** Exosomal biomarkers for the inhibition of GIC metastasis

Cancer type	Exosomal Contents	Exosome Source	Exosomal Cargo	Effect	Clinical Significance	Ref
colorectal cancer	miRNA	Cell culture fluid	miR-1827	downregulate SUCNR1	inhibit metastasis	[251]
		Serum	miR-140-3p	upregulate the expression of BCL9 and BCL2	inhibit metastasis	[155]
		Cell culture fluid	microRNA-3940-5p	upregulate TGF-β1	inhibit metastasis	[252]
		Cell culture fluid	miR-100 and miR-143	downregulate miR-100/mTOR/miR-143 axis	inhibit metastasis	[156]
	circRNA	Cell culture fluid	circFNDC3B	inhibit miR‐937‐5p and upregulate TIMP3	inhibit metastasis	[153]
		serum	circRHOBTB3	secrete outside of cells	inhibit metastasis	[154]
	lncRNA	serum	lncRNA ADAMTS9-AS1	inhibit the Wnt/β-catenin signaling pathway	inhibit metastasis	[157]
	proteins	Cell culture fluid	ANGPTL1	downregulate MMP9 level in KCs by inhibiting the JAK2-STAT3 signaling pathway	inhibit metastasis	[158]
hepatocellular cancer	miRNA	liver tissue	miR-374c-5p	activate LIMK1-Wnt/β-catenin axis	inhibit metastasis	[160]
		serum	miR-125b	block TGF-β1/SMAD Pathway, repress protein expression of SMAD2	Inhibit metastasis	[253]
		serum	microRNA-27a-3p	suppress of Golgi Membrane Protein 1	inhibit metastasis	[159]
	circRNA	serum	circ-0072089	sponge with miR-375 and upregulate MMP-16	inhibit metastasis	[161]
		Cell culture fluid	hsa_circ_0004658	activate hsa_circ_0004658/miR-499b-5p/JAM3 pathway	promote metastasis	[254]
		Plasma	hsa_circ_0051444	upregulate BAK1 and competitive bound to miR-331-4p	inhibit metastasis	[162]
gastric cancer	miRNA	Cell culture fluid	miR-29b	not mention	inhibit metastasis	[255]
	circRNA	serum	CircRNA ITCH	regulate miR-199a-5p/Klotho axis	inhibit metastasis	[165]
		Cell culture fluid	CDR1as	activate the CDR1as/miR-876-5p/GNG7 axis	inhibit metastasis	[164]
		Cell culture fluid	circRELL1	upregulate the expression of EPHB3	inhibit metastasis	[148]
pancreatic cancer	miRNA	Cell culture fluid	miR-485-3p	decrease the PAK1 expression	inhibits metastasis	[166]
		Cell culture fluid	miRNA-29b	downregulate of ROBO1 and SRGAP2	inhibits metastasis	[256]
		Cell culture fluid	miRNA-339-5p	decrease TGFBR3 levels and activate TGF-β signal	inhibits metastasis	[257]
	circRNA	Cell culture fluid	circ_0030167	activate miR-338-5p/wnt8/β-catenin axis	inhibit metastasis	[167]
		Cell culture fluid	Circ_0006790	downregulate S100A11, via CBX7-catalyzed DNA hypermethylation, bind to CBX7, MiRNA sponge for miR-144	inhibit metastasis	[168]

For CRC, Zeng et al. found that exosomal circFNDC3B can bind to miR-937-5p to upregulate TIMP3, thus inhibiting tumorigenic, metastatic, and angiogenic properties of CRC [153]. Chen et al. observed that circRHOBTB3 acts as a tumor-suppressive circRNA and inhibits CRC growth and metastasis by repressing intracellular ROS production and metabolic pathways in CRC [154]. Another study revealed that exosomal miR-140-3p overexpression suppresses CRC proliferation, β-catenin nuclear translocation, invasion, and migration via targeting BCL9 and BCL2 [155]. Babak et al. found that exosomal miR-143 and miR-100 effectively downregulate mTOR, K-RAS, HK2, and Cyclin D1 while significantly suppressing the expression of MMP9, MMP2, TWIST, and SNAIL; and upregulating p-27 expression, thus hampering CRC proliferation, migration, invasion, and metastasis [156]. Li et al. have shown that upregulation of lncRNA-ADAMTS9-AS1 can suppress β-catenin and mediate Wnt signal pathway to suppress colorectal tumorigenesis [157]. Jiang et al. demonstrated that exosomal ANGPTL1 attenuates LM and impedes vascular leakiness of PMN by downregulating MMP9 expression in KCs and restraining JAK2-STAT3 signaling pathway [158].

For HCC, exosomal microRNA-27a-3p and miR-374c-5p can respectively upregulate GOLM1 and inactivate Wnt/β-catenin pathway to suppress EMT to inhibit HCC progression [159, 160]. Besides, exosomal circ-0072088 can sponge with miR-375 and upregulate MMP-16 to suppress HCC metastasis, and exosomal hsa_circ_0051443 can bound to miR-331-3p to upregulate BAK1 which in turn suppresses HCC progression [161, 162]. Cheng et al. reported that exosomal p120ctn can inhibit expansion of liver cancer stem cells, cell proliferation, and metastasis in HCC by activating STAT3 pathway [163].

For GC, Jiang et al. found that circRNA CDR1as knockdown facilitates GC invasion and migration while the overexpression of circRCDR1as reverses aforementioned phenomena [164]. Another study reported that circRNA ITCH can regulate the miR-199a-5p/Klotho axis to inhibit GC proliferation, migration, invasion, and EMT [165]. Exosomal circRELL1 can sponge miR-637 to indirectly upregulate EPHB3 expression by modulating autophagy to block cancer cell proliferation and migration [148].

For PC, exosomal miR-485-3p suppresses PC cell invasion and migration by targeting p21-activated kinase-1 [166]. Exosomal circ_0030167 significantly reduces PC cell invasion, migration, and proliferation by regulating miR-338-5p, increasing Wif1 expression, and inhibiting the Wnt8/β-catenin pathway [167]. Exosomal hsa_circ_0006790 inhibits immune escape and metastasis in PDAC by inducing translocation of chromobox 7 [168].

### Impede production and release of exosomes

In the case of cancer metastasis, blocking exosome release to alleviate cancer migration seems like a practicable and promising approach. Ostrowsk et al. found that Rab27a and Rab27b function as MVE docking at the plasma membrane. So, silencing Rab27a and Rab27b can decrease the release of exosome [169]. For ESCRT independent pathway, silencing Rab31, caveolin-1, and flotillins which contribute to cancer chemoresistance and progression by regulating exosome secretion may exert function on decreasing CDEs production [170–172]. And RBPs (such as MVP, hnRNPA2B1, hnRNPA1, hnRNPH1, and hnRNPK) can regulate exosome generation, so blocking their expression can dwindle exosome production [173]. Vps4A can inhibit β-catenin signal pathway and EMT to suppress exosome release and restrain the proliferation and metastasis of HCC [174]. Parolini et al. demonstrated that CDEs cultured under acidic conditions exhibit more secretion and dangerous delivery activity than those cultured under physiological conditions [175]. In particular, under acidic conditions, the cancer-released exosomes are 3–8 folds compared with those cultured at pH 7.4 conditions [175]. Given this theory, proton pump inhibitors (such as NHE1 inhibitors, CA inhibitors, MCT inhibitors, and V-ATPase inhibitors), altering cellular pH, and alkalizing agents seem to be viable and potential anti-cancer strategies for metastatic patients [176–178].

Datta et al*.* revealed that Manumycin A seems to be a potential drug candidate to inhibit exosome biogenesis and release by suppressing of Raf/Ras/ERK2/1 signal pathway [179]. Asai et al. demonstrated that the restriction of SMase can decrease exosome secretion [180]. Sun et al. found that hypoxia can affect the composition of CDEs and promote metastasis [181]. Li et al. found that GW4869, the N-SMase inhibitor, can pharmacologically block exosome generation and successfully decrease exosome secretion [82, 149].

### Inhibition the interaction of exosome and recipient cell

Based on the process of exosome biogenesis, interrupting CDEs uptake seems another feasible method to avoid cancer metastasis. Sento et al. reported that heparin can remarkably decrease the uptake of CDEs to abrogate tumor progression and metastasis [182]. Hoshino et al. revealed that 4175-LuT exosomes preincubate with HYD-1, and ITGβ5 knockdown in BxPC-3-LiT exosomes and target ITGα6β4 and αvβ5 can respectively diminish exosome uptake in the liver and lung to block cancer metastases [18]. Gong et al. reported that low-pH and hypoxia treatment could significantly evaluate exosome uptake effectiveness by changing the lipid composition in exosome membrane [183]. So, the aforementioned proton pump inhibitors and alkalizing agents can alleviate exosome uptake by reversing the hypoxia and low-pH environment [184].

### Exosomes as the drug delivery system in cancer therapy

Given the exosomal hallmarks: ①specificity, safety, and stability which protected by phospholipid bilayer membrane, ②exosome is distributed in all biological fluids which allow exosome to arrive at any site, ③low immune response, ④able to pass the Blood–Brain Barrier, ⑤low systemic toxicity [185]. Harnessing exosomes as the desirable tool to deliver drugs to target cancer sites seems a feasible method. Drugs can directly or indirectly load into exosomes, including through coculture, electroporation, liposomes, sensitive fusogenic peptide, sonication, cationic lipids, and metal–organic nanoparticles coated with exosome [186]. You et al. reported that exosomes can transfer L-PGDS to cancer cells to inhibit gastric cancer progression [187]. Besides, Pascucci et al. found that the exosomes isolated from paclitaxel co-cultured with MSCs exhibit strong anti-tumor activity [188].

## Conclusions and future perspectives

Within the past decades, the detection methods for GIC have emerged in an endless stream. In particular, liquid biopsy relies on the advantages of minimal invasiveness, being readily available, and allowing real-time monitoring to hew out a new avenue for GIC pre-metastasis detection. Especially with the proposal of exosome, liquid biopsy reaches the golden period. Exosome gained extensive attention, reshaping our understanding of cancer biology and anti-cancer strategies, and providing new biomarkers for cancer diagnosis. The current studies consistently demonstrate that CDEs play an indispensable role in the biogenesis of GIC invasion and metastasis. Exosomes can not only participate in precancerous processes such as EMT, PMN formation, and immune escape, but also establish a suitable microenvironment for GIC metastasis by rendering exosomes to communicate with pending sites and shuttling proteins and nucleic acids into distant organs.

However, challenges and opportunities coexist in applying exosomes as the biomarker for GIC metastasis detection. There are still several problems to be addressed which may imply some worthwhile directions for future studies. First, there is a lack of standard methodology and standardized criteria for high-purity exosome separation, because different isolation methods may result in high heterogeneity. There is an urgent need for exosome isolation, examination, and production to develop a unified standardization. Second, distinct bioactive molecules contained in exosomes have different functions in promoting cancer metastasis. Although the aforementioned text briefly listed the effects of different components on cancer metastasis, the accurate function of exosome cargo and their association is ethereal. It is essential to identify which contain exosome is responsible for which part and their link when utilizing exosomes in metastatic cancer screening. There are some pivotal questions remain to be answered ①The potentiality of conversing biomarkers of liquid biopsy into clinical practice are still hindered by several limitation. How to transform scientific achievements into clinical applications? ②How to deliver the drug-incubated exosomes into ideal site to inhibit cancer metastasis? ③How to precisely reverse exosome-triggered immune evasion? There is a long way to go before we can convert carcinogens into cancer treatments.

## Data Availability

Not applicable.

## Abbreviations

GIC: Gastrointestinal cancer
CDEs: Cancer-derived exosomes
CRDs: Cancer-related deaths
CRC: Colorectal cancer
PC: Pancreatic cancer
CT: Computed tomography
EVs: Extracellular vesicles
EMT: Epithelial-mesenchymal transition
PMN: Pre-metastatic niche
ECM: Extracellular matrix
ITGs: Integrins
ESE: Early-sorting endosome
LSEs: Late-sorting endosomes
ILVs: Intraluminal vesicles
MVBs: Multivesicular bodies
ESCRT: Endosomal sorting complexes required for transport
miRNAs: MicroRNAs
circRNA: circular RNAs
lncRNAs: Long non-coding RNAs
MIF: Migration inhibitory factor
PDAC: Pancreatic ductal adenocarcinoma
KCs: Kupffer cells
TGFβ: Transforming growth factor beta
CAFs: Cancer-associated fibroblasts
TME: Tumor microenvironment
IGF: Insulin-like growth factor
EGF: Epidermal growth factor
HIF-1α: Hypoxia-inducible factor 1 alpha
NK: Natural killer
Tregs: Regulatory T cells
TAMs: Tumor-associated macrophages
PD-L1: Program death ligand 1
PD1: Programmed cell death 1
TNM: Tumor-node-metastasis
CEA: Carcinoembryonic antigen
CA19-9: Carbohydrate antigen
ROC: Receiver operating characteristic
AUC: Area under the ROC curve
FMNL2: Formin-like 2
LM: Liver metastasis
OS: Overall survival
ZO-1: Zonula occlusion 1
SNX2: Sorting nexin 2
PCAT1: Prostate cancer associated transcript 1
MALAT1: Metastases-associated lung adenocarcinoma transcript 1
CTLA-4: Cytotoxic T lymphocyte antigen 4
VM: Vasculogenic mimicry
HuR: Human antigen R
SRSF3: Ser/Arg-rich splicing factor 3
PDCD4: Programmed cell death 4
ASPH: Aspartate β-hydroxylase
